# A Crack‐Based One‐Dimensional Microspheres Array Enables Thermal–Mechanical Decoupled Dual‐Functional Sensing

**DOI:** 10.1002/advs.75452

**Published:** 2026-04-27

**Authors:** Wanqing Xu, Hongyi Tu, Zehao Wang, Tianyu Zhu, Chao Wang, Min Chen, Lan Shi, Limin Wu

**Affiliations:** ^1^ College of Smart Materials and Future Energy State Key Laboratory of Coatings for Advanced Equipment Advanced Coatings Research Center of Ministry of Education of China Fudan University Shanghai China

**Keywords:** bioinspired microspheres array, crack‐based sensing, flexible electronics, multimodal sensing soft materials, structural signal modulation, thermal–mechanical decoupling, ultralow‐strain detection

## Abstract

Strain sensors in soft‐material and flexible‐electronic systems face a fundamental challenge: isotropic thermal expansion can generate parasitic signals that obscure true mechanical deformation. This issue is particularly severe in crack‐based sensors, whose extreme morphological sensitivity causes thermally driven crack evolution to interfere with strain transduction. Inspired by the bead‐chain morphology of *Nostoc*, we introduce a crack‐based one‐dimensional microspheres array (COMA) that stabilizes crack geometry under isotropic expansion, enabling a predictable, monotonic thermal response from which true strain can be accurately extracted. Conductive polyaniline (PANI)@ polystyrene (PS) microspheres unidirectionally assembled into grooved elastomers form discrete, crack‐like junctions that convert ultralow mechanical deformation (≤0.5%) into pronounced electrical signals. The COMA sensor exhibits a stable thermal response of 75.2%°C^−1^ (20°C–60°C) and, when integrated with a multilayer perceptron model, classifies four operational states of a pouch cell with 97.0% accuracy. This bioinspired and structurally guided strategy establishes a general approach for constructing multimodal, thermomechanically stable, intelligent flexible sensors.

## Graphical abstract

Inspired by *Nostoc*, a crack‐based one‐dimensional microspheres array (COMA) sensor is developed, which stabilizes crack geometry under isotropic expansion, enabling a predictable, monotonic thermal response from which true strain can be accurately extracted. The COMA sensor exhibits high sensitivity at ultralow deformation (gauge factor up to 89) and a distinct thermal response (75.2%°C^−1^ from 20°C–60°C).

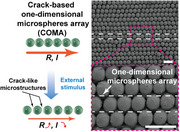

## 1 Introduction

Strain sensors used in soft‐material and flexible‐electronic systems often experience coupled mechanical and thermal stimuli, complicating the separation of true deformation from temperature‐induced strain [1, 2]. Isotropic thermal expansion in polymer substrates can generate parasitic strain signals comparable to mechanical inputs, undermining high‐fidelity sensing in applications ranging from wearable analytics to soft robotics and energy devices [3, 4, 5]. Across strain‐dependent transduction mechanisms—including capacitive [6, 7], piezoresistive [8. 9], and crack‐mediated systems [10, 11]—thermal–mechanical interference remains a pervasive obstacle, and crack‐based sensors are particularly vulnerable owing to the extreme thermal sensitivity of crack opening.

Because of this heightened susceptibility, crack‐based sensors serve as a representative platform for exploring mechanically guided routes to high‐sensitivity strain detection [12]. Prior studies have introduced engineered microstructures on flexible substrates—such as patterned stiff layers [13, 14], line‐oriented surface reliefs [15, 16], and meandered conductive films [17]—to promote ordered channel cracking and suppress uncontrolled networks under uniaxial loading. These approaches improve reproducibility and tunability but inherently assume a directional strain field and offer no means to separate mechanical strain from isotropic thermal expansion. Thermal‐induced isotropic strain can rotate, distort, or coalesce cracks [5, 18, 19], transforming well‐aligned channels into disordered networks and degrading sensitivity, stability, and long‐term reliability. Critically, no existing strategy provides a structural control of crack morphology that renders thermal responses sufficiently stable and predictable to enable accurate microscale separation of mechanical strain from thermal effects.

To address this challenge, we developed a crack‐based one‐dimensional microspheres array (COMA) sensor inspired by the bead‐chain morphology of *Nostoc*. Conductive microspheres are unidirectionally assembled into grooved elastomer substrates, converting planar cracks into discrete point‐like junctions. These junctions respond directionally to mechanical strain, while the stabilized architecture ensures thermally induced changes are monotonic and reproducible, allowing accurate strain extraction at any temperature. The COMA sensor exhibits high sensitivity at ultralow deformation (≤0.5%; gauge factor up to 89) and a distinct thermal response (75.2%°C^−1^ from 20°C–60°C). Leveraging this dual‐mode capability, we integrated the COMA sensor into a pouch cell, where a multilayer perceptron (MLP) model classified four battery states with 97.0% accuracy.

## 2 Results

### 2.1 Design and Fabrication

When the strain exceeds the critical strain value of the materials, the cracks form to release the stress and rebuild the local microstructure [20, 21]. Based on this principle, when such cracks form within conductive thin films (e.g., metal layers), they disrupt electrical continuity and result in a pronounced increase in resistance [10, 19]. Thus, the crack‐based strain sensor can be prepared through the mechanism mentioned above. In the unstrained state, the channel cracks in conductive films remain tightly closed [13], keeping the sensor in a low‐resistance baseline condition. On the other hand, as the applied strain increases, these cracks gradually open, disrupting the conductive path and bringing the sensor to a high‐resistance state [8]. While many works have attempted to obtain strain sensors with specific performance through controllable crack design [15, 22], it should be noticed that the thermal expansion of the polymer substrate induces additional isotropic strain, which may disrupt the formation of channel cracks and make it fail to perform sensing. As a result, it is a challenge for conventional crack‐based sensors to resist temperature changes, let alone be compatible with temperature‐sensing functions.

**FIGURE 1:**
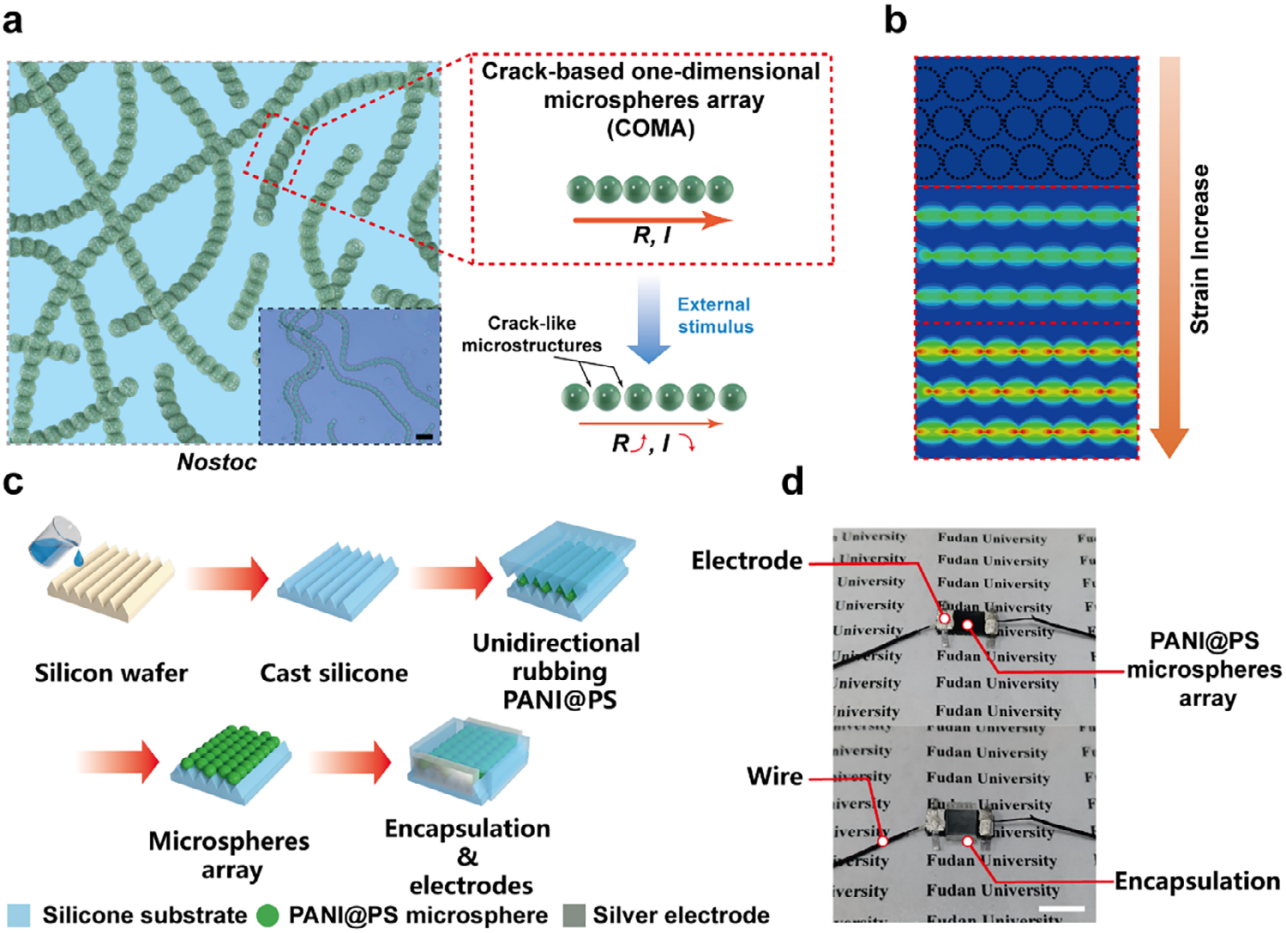
Schematic illustration of the COMA sensor. (a) The optical graph and the schematic diagram of the morphology of *Nostoc* and the conception of a crack‐based one‐dimensional microspheres array (COMA). Scale bar, 10 µm. (b) Simulation results of the COMA under various strains. (c) Fabrication process of the COMA sensor. (d) The optical graph of the COMA sensor. Scale bar, 1 cm.

To overcome the limitations of random crack propagation, a biomimetic approach was adopted to achieve ordered, controllable crack‐like microstructures. Inspired by *Nostoc*, a filamentous cyanobacterium composed of chains of spherical cells connected end‐to‐end [23], we designed a crack‐based sensor architecture mimicking its bead‐like morphology (Figure 1a). Based on the unique morphology, we conceive a new type of crack‐based sensor based on a one‐dimensional conductive microspheres array. To detect the response of the one‐dimensional conductive microspheres array with respect to different stimuli, a simplified 2D model was used to explore the strain via Finite Element Method (FEM) analysis. According to the FEM analysis results, upon the external deformation, the strain is predominantly localized at the contact parts of the microspheres (Figure 1b). Such localization of strain indicates that resistance modulation in the COMA sensor primarily originates from variations in contact resistance between adjacent microspheres. The microspheres tend to move away from each other, and the gaps between them function as microcrack structures, which results in the increase of resistance.

To realize the aforementioned conception, the fabrication process is schematically illustrated in Figure 1c. Briefly, the groove array silicone substrate was first prepared through the drop‐cast process on a customized silicon wafer. Then, two identical groove array silicone substrates were placed face‐to‐face along the longitudinal structure. The one‐dimensional conductive microspheres array was subsequently obtained by unidirectional rubbing of polyaniline (PANI)@ polystyrene (PS) conductive microspheres along the longitudinal path [24]. Finally, silver ink was applied to form the electrodes, and a thin silicone encapsulation layer was added to protect the sensor from external mechanical disturbance. The optical graph of the COMA sensor can be visually seen in Figure 1d.

**FIGURE 2:**
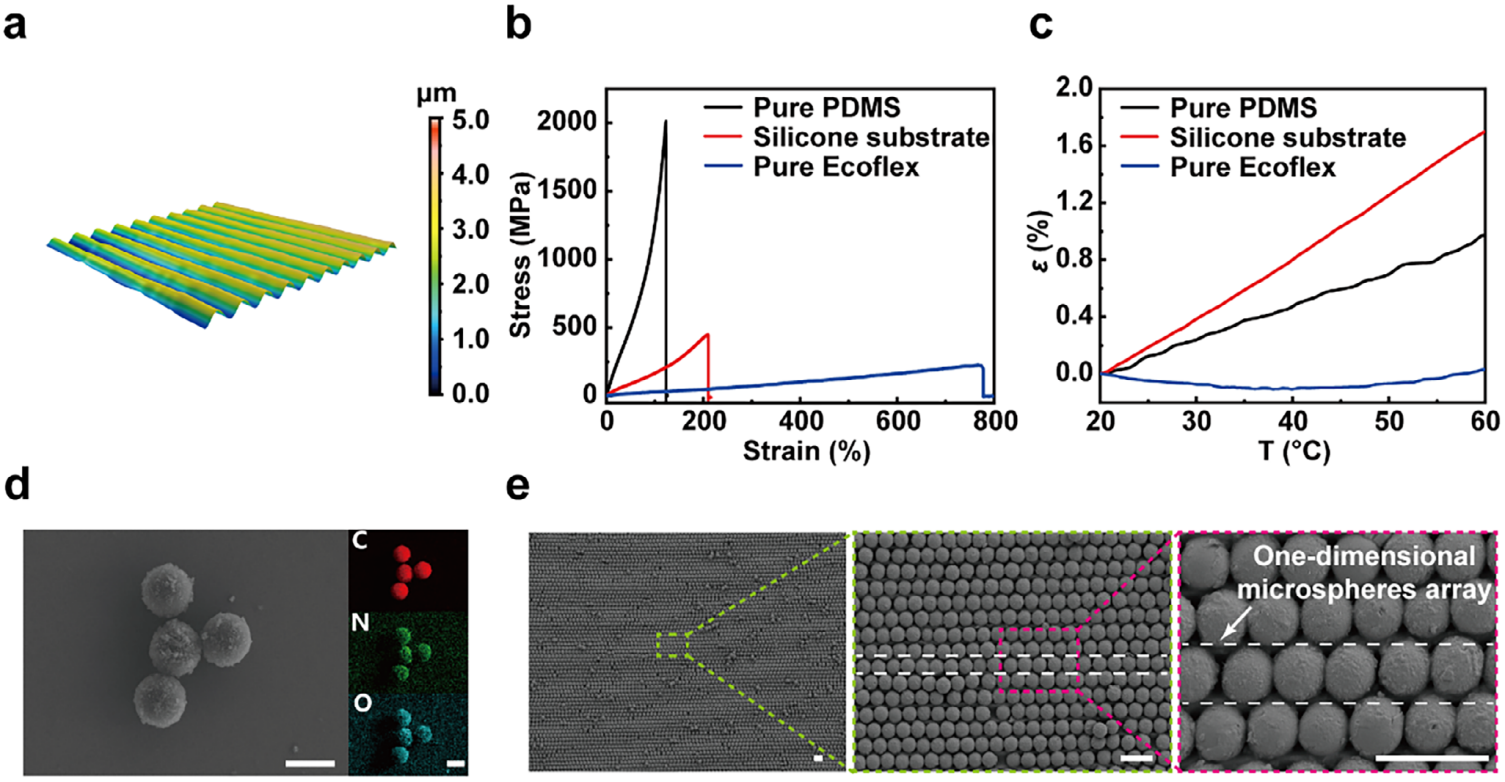
The characteristics of the COMA sensor. (a) The three‐dimensional graph of the groove array silicone substrate. (b) The stress–strain curves of pure PDMS film, silicone substrate, and pure Ecoflex film. (c) The thermal expansion amount of pure PDMS film, silicone substrate, and pure Ecoflex film within the temperature range of 20°C–60°C. (d) The SEM image and the EDS result of the PANI@PS microspheres. Scale bar, 10 µm. (e) The SEM images of the top view of the COMA. Scale bar, 20 µm.

Figure S1 presents the scanning electron microscope (SEM) images of the silicone substrate, showing that the surface of the substrate is a regular array of grooves. This structural feature can be further confirmed by the three‐dimensional graph of laser scanning confocal microscope (LSCM), as shown in Figure 2a. The silicone substrate is composed of a mixture of polydimethylsiloxane (PDMS) and Ecoflex in a mass ratio of 1:1, which takes into consideration the modulus and elongation of both materials (Figure 2b). Figure 2c presents the thermal expansion behavior of PDMS, Ecoflex, and the silicone substrate, and their coefficient of thermal expansion (CTE) were calculated, respectively. The results show that within the temperature range of 20°C–60°C, the CTE of the silicone substrate is up to 420 ppm/K, which is much greater than PDMS (∼240 ppm/K) or Ecoflex (∼8 ppm/K). The enhanced thermal expansivity likely originates from increased amorphous regions in the PDMS/Ecoflex blend, which improves its compliance and thermal responsiveness [25].

To obtain the conductive microspheres, monodispersed PS microspheres with a diameter of 10 µm (Figure S2) were used as the core, and further prepared a PANI conductive shell layer through in situ polymerization of aniline (ANI) [26, 27]. As shown in Figure S3a, PS microspheres were visually coated by PANI, and the PANI@PS microspheres maintained monodispersed (Figure S3b). The energy dispersive X‐ray spectroscopy (EDS) mapping results further verify that the PANI coating on PS microspheres should be uniform (Figure 2d). Moreover, Fourier transform infrared (FT‐IR) and Raman spectra are performed to verify the successful coating of PANI, as shown in Figure S4a,b, respectively. In Figure S4a, the characteristic peaks of the FT‐IR spectrum at 1572, 1484, and 1297 cm^−1^ are clearly observed both in PANI and the PANI@PS microspheres, which are assigned to the stretching vibrations of the quinonoid rings, the stretching vibrations of C═C on the benzenoid ring, and the stretching vibrations of C─N, respectively [28, 29, 30]. In Figure S4b, the characteristic peaks of Raman spectrum at 1164, 1496, 1556, and 1601 cm^−1^ are assigned to the C─H bending vibration of the benzenoid ring, the C═N stretching vibration of the quinoid rings, the C═C stretching vibration of quinoid rings and the C─C stretching vibration of benzenoid rings in PANI, respectively [28, 31, 32]. To determine the coating amount, the results of thermogravimetric analysis (TGA) are presented in Figure S5, showing that the mass proportion of PANI in PANI@PS microspheres is approximately 10%. As a result, this PANI shell layer endows the PANI@PS microspheres with excellent electrical conductivity (Figure S6), indicating the potential for constructing conductive pathways.

After the unidirectional rubbing process, the one‐dimensional conductive microspheres array was obtained. As shown in Figure 2e, all the PANI@PS microspheres are arranged in the grooves, which forming one‐dimensional conductive pathways. As all the grooves are arranged in parallel, the COMA sensor is equivalent to each conductive path being connected in parallel. Moreover, it should be noted that only a silicone substrate with a microstructure of grooves on its surface can form the one‐dimensional conductive pathway. This is because the grooves physically constrain the arrangement of the microspheres, which enables the microspheres to be closely arranged and not easily fall off. As the optical graphs present in Figure S7, PANI@PS microspheres were attempted to unidirectionally rub onto the silicone substrates with and without the microstructure of grooves. Remarkably, the PANI@PS microspheres failed to adhere to the silicone substrate without the grooves, underscoring the necessity of the groove microstructure for stable alignment. In addition, since the ridges of the groove separate the one‐dimensional conductive pathway, the COMA sensor is conductive in the direction parallel to the groove, while the resistance is high in the direction perpendicular to the groove. This anisotropic conductivity is experimentally verified in Figure S8, indicating that the conductive pathway is exactly one‐dimensional. The well‐defined grooves not only guide the alignment of microspheres but also enhance interfacial adhesion, ensuring robust and reproducible fabrication.

### 2.2 Working Mechanism

As shown in Figure 2e, the COMA sensor is composed of a series of one‐dimensional conductive pathways, which are constructed by PANI@PS microspheres. Notably, only the pathways aligned parallel to the grooves contribute significantly to electrical conduction, whereas perpendicular routes show negligible current flow, as confirmed by Figure S8. Since the contact points between adjacent microspheres act as crack‐like microstructures, a tiny strain can affect the integrity of the conductive path and output a resistance signal, which ensures the high sensitivity of the COMA sensor. Owing to its anisotropic architecture, thermal expansion primarily affects the longitudinal direction of the microsphere array, allowing the COMA sensor to simultaneously detect temperature‐induced strain and external mechanical deformation.

For the sake of discussion, it is rational to regard the entire COMA sensor as a series of parallel resistors. The model of the COMA sensor is schematically illustrated in Figure S9a, and the detailed theoretical model and derivations can be seen in Note S1. Briefly, the COMA can be regarded as multiple rows of conductive pathways, which are composed of PANI@PS microspheres. As a result, the ideal connection state of the resistors of the COMA sensor is shown in Figure S9b.

According to the equivalent circuit model (Note S1), the resistance–strain relationship follows an exponential form, consistent with the observed exponential dependence in the experiment.

(1) $$\frac{\Delta R}{R_{0}}=\frac{R\left(\varepsilon \right)-R_{0}}{R_{0}}\;\approx e^{A\varepsilon }-1$$

where ε is the strain of the COMA sensor, and *A* is an exponential factor related to the property of the sensing system.

This exponential response arises from the rapid tunneling resistance change at interparticle contacts as the microcracks open. Moreover, it should be noted that the silicone substrate of the COMA sensor undergoes thermal expansion upon temperature variation. This slight deformation can also be detectable by the COMA sensor. According to Equation (1), the output signal of temperature sensing should be as follows:

(2) $$\begin{array}{c} \frac{\Delta R}{R_{0}}=\frac{R\left(\varepsilon \right)-R_{0}}{R_{0}}=e^{A\varepsilon }-1=e^{A\cdot CTE\cdot \left(T-T_{0}\right)}-1 \end{array}$$

This theoretical framework suggests that the COMA sensor can intrinsically integrate thermal and mechanical stimuli, providing a unified signal output without additional calibration. In summary, the COMA sensor's unique structural design, characterized by anisotropic conductivity and parallel resistor networks, theoretically enables dual functions of strain sensing and temperature sensing.

### 2.3 Sensing Performance

#### 2.3.1 Performance of Strain Sensing

Based on the sensing mechanism shown above, the COMA sensor is expected to achieve high‐sensitivity strain sensing. To accurately characterize its performance, the commercial polyethylene terephthalate (PET) tape was adhered underneath the COMA sensor, which can enhance the structural integrity of the sensing system while preserving its sensitivity. As shown in Figure S10a, the PET tape has a high elastic modulus up to 3.1 GPa. Furthermore, the PET tape complies with Hooke's law well when the strain is less than 1% (Figure S10b). As a result, the PET tape effectively stabilizes the COMA sensor during the tensile testing process without compromising its strain‐sensing performance.

**FIGURE 3:**
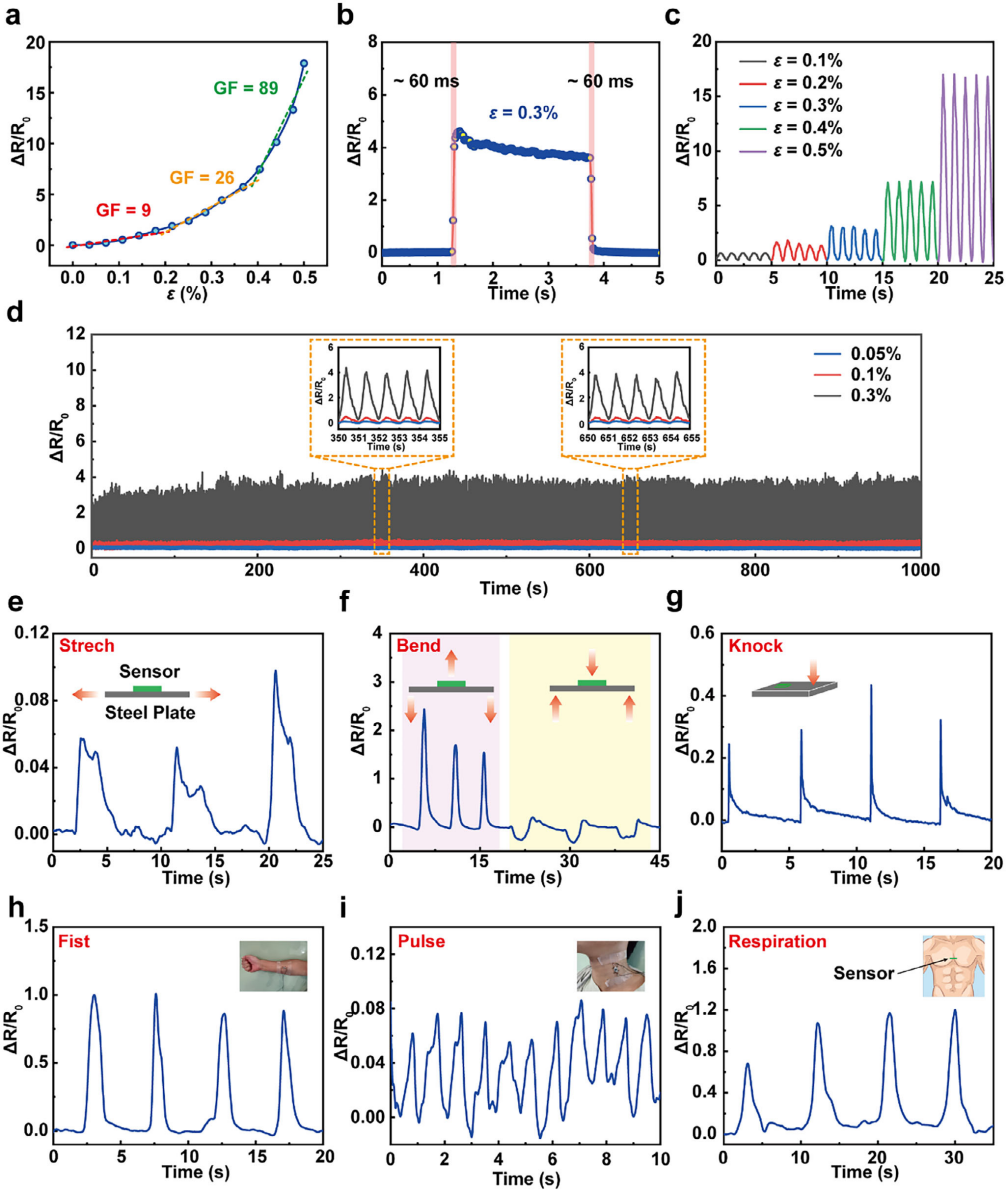
The performance of strain sensing of the COMA sensor. (a) Normalized resistance changes and sensitivity of the COMA sensor in strain sensing. (b) Response time and relaxation time. (c) Normalized resistance changes in response to the different strains. (d) Cyclic response of the COMA sensor up to 1000 cycles under the strain of 0.05%, 0.1%, and 0.3%, respectively. (e–g) Applications of the sensor in monitoring inconspicuous deformations of the steel plate, including stretching, bending, and knocking. (h–j) Applications of the sensor in physiological signals and body movements, including fisting, pulse, and respiration.

The strain‐dependent electrical response of the COMA sensor is illustrated in Figure 3a. Owing to the gaps between the PANI@PS microspheres, which function as the microcrack structures, the COMA sensor realizes high sensitivity strain sensing within a strain of 0%–0.5%. The sensitivity of the sensor can be quantified by gauge factor (GF), which can be defined as:

(3) $$GF=\frac{\Delta R/R_{0}}{\varepsilon }$$

where Δ*R* is the change in resistance, *R*_0_ is the initial resistance, and ε is the applied strain.

By fitting the normalized resistance curve, the slope of the curve increases in three regions of 0%–0.2%, 0.2%–0.4%, and 0.4%–0.5%. The GF in different regions was calculated, which were 9, 26, and 89, respectively, as shown in Figure 3a. Moreover, fitting lines for logarithmic normalized resistance are shown in Figure S11. The results show that when the strain is above 0.1%, the normalized resistance exhibits an exponential correlation with strain, which is consistent with Equation (1). For the strain below 0.1%, it is supposed that the normalized resistance may be influenced by the *R_s_*, while it still maintains an exponential correlation with strain. Additionally, the normalized resistances of the sensor are also generally consistent across three stretching processes with a maximum strain of 0.2% (Figure S12), which verifies the consistency during strain sensing.

Response time serves as a critical parameter that enables sensors to effectively detect dynamic signals. The COMA sensor demonstrates rapid response and relaxation times of approximately 60 ms during loading and unloading (Figure 3b). The fast response of the COMA sensor guarantees the accuracy and effectiveness of real‐time dynamic detection. Figure 3c shows the corresponding output signals when the COMA sensor responds to the different dynamic external strains, indicating the fast response and high sensitivity of the sensor. To assess the long‐term usage potential of the COMA sensor, cyclic loads with different strains were applied. Figure 3d shows the signal change of the COMA sensor under the strain of 0.05%, 0.1% and 0.3% relatively during cyclic loading‐unloading for 1000 cycles. The results ensure the durability and stability of the COMA sensor during long‐term use. Moreover, the SEM images of the COMA sensor before and after the cyclic mechanical loading are shown in Figure S13. The image shown in Figure S13b indicates that large‐strain stretching slightly affects the uniformity of the microsphere array on the surface of the COMA sensor and causes slight signal drift. These results collectively demonstrate that the COMA sensor combines ultrahigh sensitivity, fast response, and long‐term operational stability—key attributes for real‐world applications in subtle strain detection.

The excellent performance of the COMA sensor enables it to monitor the subtle strain of objects. To prove this, the COMA sensor was affixed on the steel plate with commercial tape. When the volunteer manually stretched the steel plate, the sensor generated an obvious signal output, indicating the slight deformation of the steel plate, which is undetectable through human perception (Figure 3e). Furthermore, the volunteer attempted to bend the steel plate in both directions, and the output signals are shown in Figure 3f. In the first stage, the bending of the steel plate tends to stretch the COMA sensor, causing a corresponding increase of resistance. In the subsequent stage, the bending of the steel plate in the opposite direction tends to compress the sensor, and the resistance of the sensor decreases correspondingly. These responses highlight the sensor's ability to distinguish between tensile and compressive strains. As a strain sensor, the COMA sensor also has the potential to monitor vibration. As shown in Figure 3g, the volunteer knocked the steel plate, which is approximately 5 cm away from the sensor, the COMA sensor not only detects the occurrence of vibration but also continuously monitors its attenuation process.

Besides rigid objects, the COMA sensor can be further used on the human body and monitor physiological and movement signals, including muscle activity, cardiovascular signals, and respiration. As shown in Figure 3h, the COMA sensor was adhered to the inner side of the forearm and successfully recorded the contraction of the forearm muscles when the volunteer made a fist. When the sensor was positioned near the carotid artery, it captured the volunteer's pulse waveform signal (Figure 3i). When the sensor was attached to the chest, it could detect the volunteer's respiratory patterns (Figure 3j). These results show the versatility of the COMA sensor in health monitoring.

#### 2.3.2 Performance of Temperature Sensing

As the fact that elastic polymers have a higher CTE compared to other materials, they are more susceptible to thermal expansion. As discussed in the working mechanism and Note S1, the COMA sensor, which is constructed on the polymer substrate, can monitor the thermal expansion of the silicone substrate in response to ambient temperature changes. On one hand, this slight deformation is reflected in the COMA sensor's resistance variations, realizing the function of temperature sensing. On the other hand, thermal expansion only slightly increases the spacing between adjacent one‐dimensional arrays without altering the whole conductive pathways—thus decoupling the effects of temperature and strain.

Moreover, the differential scanning calorimetry (DSC) thermograms confirmed that no phase transformation occurs in the silicone substrate and the PANI@PS microspheres within the temperature range of 20°C–60°C (Figure S14), indicating that the deformation of the COMA sensor is solely due to thermal expansion. Thus, the COMA sensor has the capability to realize temperature sensing owing to the slight strain induced by the thermal expansion of the silicone substrate.

**FIGURE 4:**
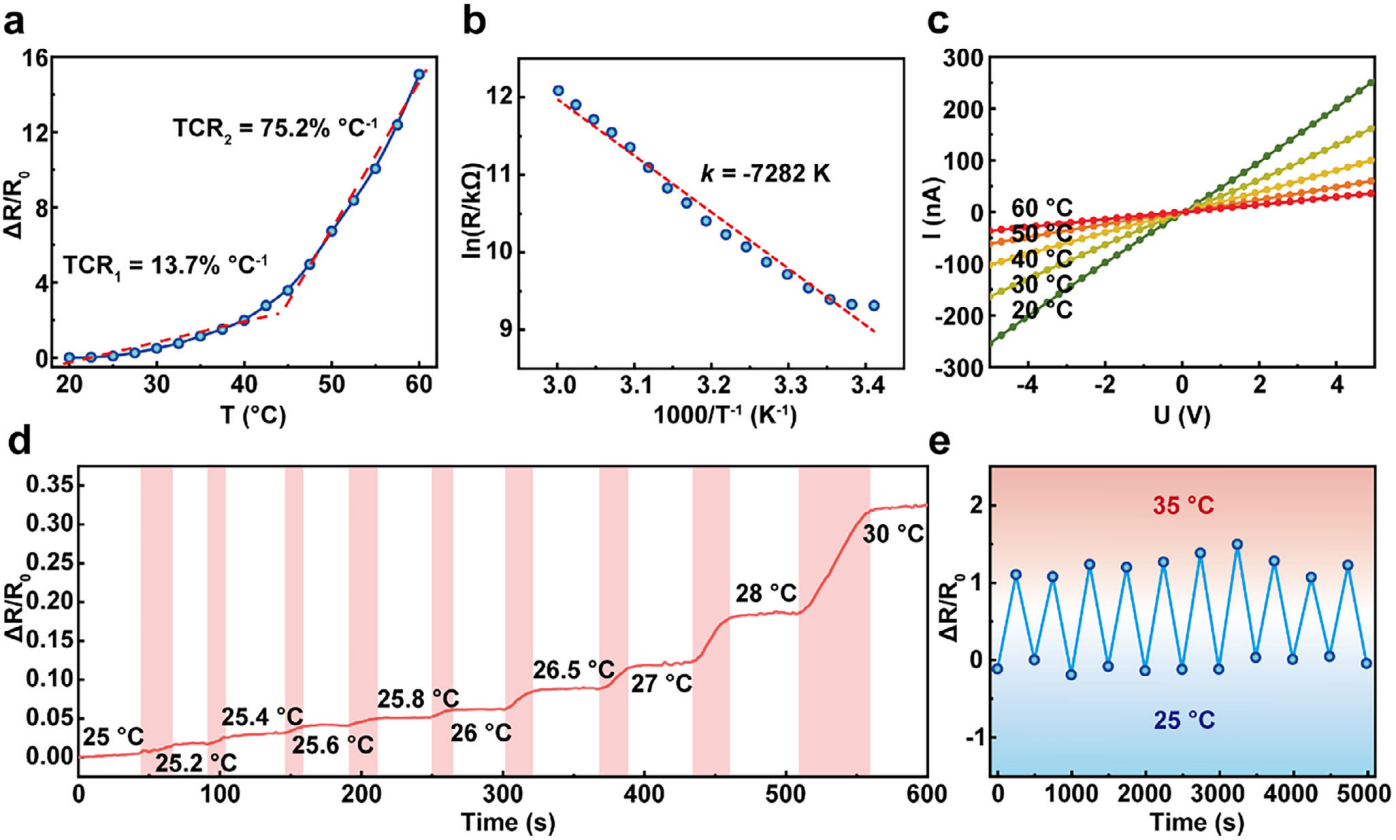
The performance of temperature sensing of the COMA sensor. (a) Normalized resistance changes and sensitivity of the COMA sensor in temperature sensing. (b) Arrhenius plots of the COMA sensor. (c) Current−voltage curves of the COMA sensor at different temperatures. (d) Temperature change detected by the COMA sensor within the range of 25°C–30°C. (e) Sensor response to 10 heating and cooling repeated cycles between 25°C and 35°C.

The temperature‐dependent resistance response of the COMA sensor is presented in Figure 4a. The sensitivity of the sensor can be quantified by the temperature coefficient of resistance (TCR), which is defined as:

(4) $$\begin{array}{c} TCR=\frac{\Delta R/R_{0}}{\Delta T} \end{array}$$

By fitting the normalized resistance, the TCR of the COMA sensor was calculated to be 13.7%°C^−1^ in the temperature range of 20°C–45°C, and 75.2%°C^−1^ in the temperature range of 45°C–60°C. Compared with the previous reported temperature‐sensitive flexible sensors, the COMA sensor has superior sensitivity and an extended detection range (Table S1 and Figure S15). Furthermore, over the entire sensing range of 20°C–60°C, the Arrhenius plots of the COMA sensor demonstrate its excellent linearity and exhibit a high thermal index *k* of −7282 K (Figure 4b), highlighting its reliability in temperature monitoring applications. Additionally, the normalized resistances of the sensor are also generally consistent across three heating processes within the range of 25°C–35°C (Figure S16), which verifies the consistency during temperature sensing.

To further characterize the temperature sensing performance of the COMA sensor, current‐voltage curves of the sensor under different temperature is illustrated in Figure 4c. The results show that the COMA sensor behaves as a pure resistance throughout the entire temperature sensing range. Moreover, the result of a temperature programmed test within a range of 25°C–30°C is shown in Figure 4d, indicating that the COMA sensor can capture varying degrees of tiny temperature fluctuations and the limit of detection (LOD) is as low as 0.2°C. Notably, dynamic measurement results of the COMA sensor to the heating and cooling processes between 25°C and 35°C for 10 repeated cycles are shown in Figure 4e. Furthermore, as shown in Figure S17, the maximum hysteresis of the COMA sensor is only 5.6% within the range of 25°C–35°C. These results ensure the stability of the sensor during cyclic thermal processes. The repeatability of the COMA sensor is characterized as shown in Figure S18, indicating that after 100 heating cycles, the maximum deviation of the COMA sensor is 13.7%. Such reproducible thermal responses underscore the COMA sensor's reliability for long‐term and repeated temperature monitoring.

The low‐temperature sensing performance of the COMA sensor is further characterized in Figure S19. Under the ambient condition of 25°C, a drop of ice water was gently applied onto the upper surface of the COMA sensor. The gravity of the droplet induced a slight deformation, leading to an initial increase in resistance followed by a rapid decrease due to the thermal contraction of the silicone substrate. As the drop of the ice water was removed by a piece of blotting paper, the resistance returned to its initial state, demonstrating the sensor's recovery capability.

The reliability of sensors in diverse environments is of great significance. Figure S20 shows the resistance changes of the COMA sensor during the stretching process at different adjacent temperatures. The results verify that even facing with coupled stimuli, the decoupling of temperature and strain can be achieved. The COMA sensor can be partly modified into two parts, with one shielded from the effect of external strain by applying a high‐modulus substrate, and the other remains unchanged. Thus, the output signal acquired from the former part is only related to temperature, while the output signal acquired from the latter part is determined by both temperature and strain. According to Figure S20, based on the measured temperature, the corresponding strain curve can be matched, which enables to achieve the actual strain. In order to evaluate the long‐term sensing performance, the COMA sensor was subjected to continuous heating at different constant temperatures for 1200 s. The results in Figure S21 show that even after long heating, the fluctuation of resistance remains less than 3.5%, indicating the excellent temperature sensing reliability of the COMA sensor. Moreover, Figure S13 indicates that the morphology of the one‐dimensional conductive microspheres array is largely maintained after long heating. The above characterizations confirm that the COMA sensor has the potential for long‐term temperature monitoring.

### 2.4 Application for Battery State Monitoring System

With the rapid advancement of wearable technology, a wide range of devices have emerged, capable of functions such as health monitoring and movement detection. These wearable devices typically rely on batteries for energy supply. Currently, various types of pouch cells are widely used in wearable systems due to their high battery energy density and portability [33, 34]. Notably, pouch cells are renowned for their safety performance, as they only swell facing with failures [35]. However, prolonged cycling can induce subtle swelling in pouch cells [36], which may compromise safety and operational reliability in wearable electronics. As a result, it is necessary to continuously monitor the states of the pouch cell for operational safety and performance.

**FIGURE 5:**
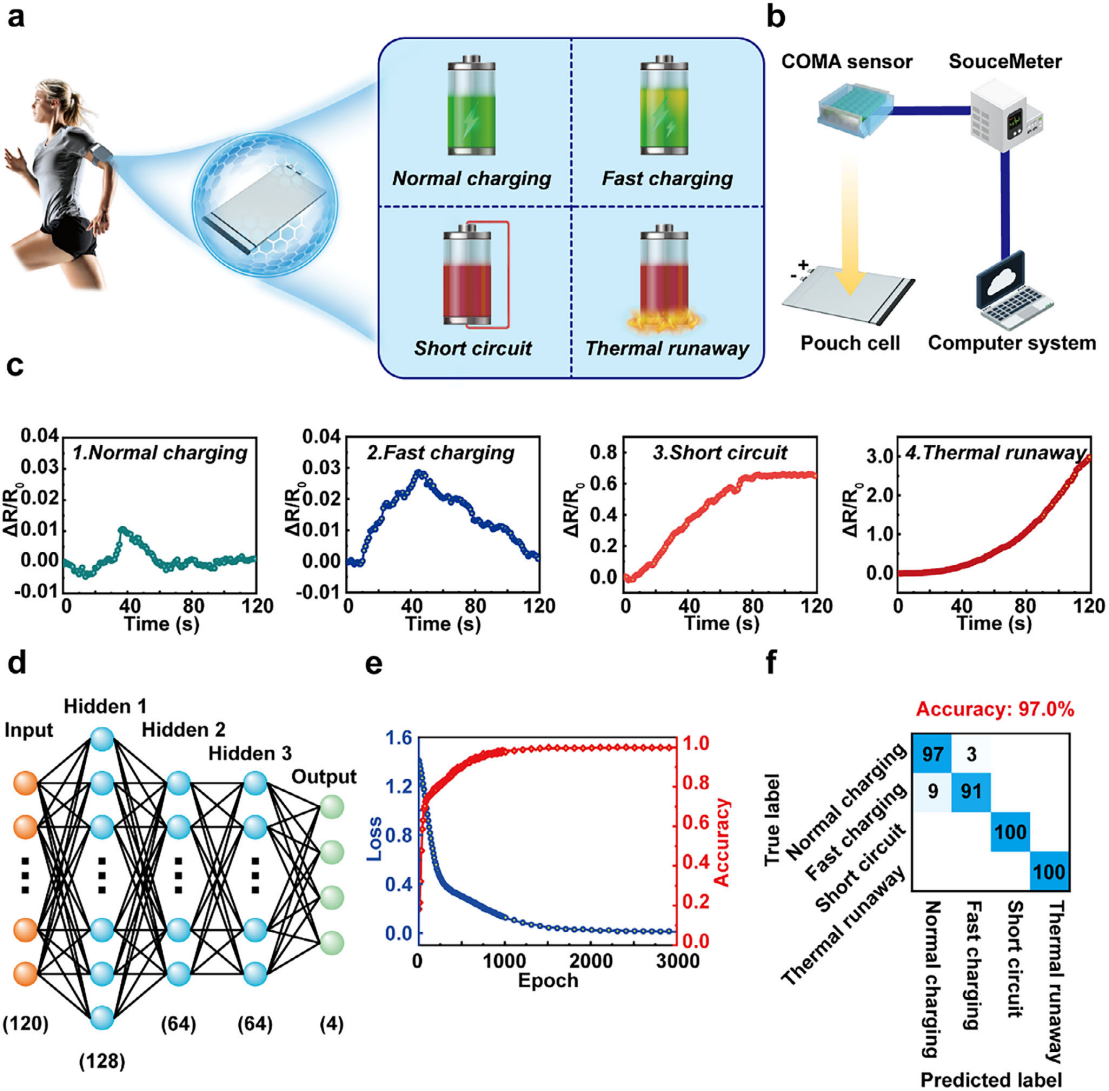
Battery state monitoring based on the COMA sensor. (a) The schematic diagram of the pouch cell used for wearable devices and the four operational states of the cell. (b) The schematic diagram of the application for the battery state monitoring system. (c) Normalized resistance changes of the COMA sensor under four states of the cell. (d) The structure diagram of an artificial neural network (ANN). (e) The tendency of accuracy and loss on the training set during the training process for 3000 epochs. (f) The confusion matrix of the classification of four states of the pouch cell.

Notably, pouch cells display characteristic thermal and mechanical signatures under different operational or failure modes—such as temperature elevation and volumetric expansion [37], which can be precisely captured by the COMA sensor. Herein, as shown in Figure 5a, four operational states of the pouch cell are analyzed in detail. (1) Normal charge. The cell is charged under ideal conditions, including rated voltage and current. (2) Fast charge. To improve charging efficiency, the cell can be charged at a higher voltage or current at the expense of its long‐term performance. (3) Short circuit. Direct contact of the positive and negative terminals of the cell, releasing excessive heat according to Joule's law [38]. (4)Thermal runaway. The cell experiences a sudden temperature rise due to abnormal factors such as high temperature or leakage [39]. Based on these states, a COMA sensor was applied onto the surface of a pouch cell to construct a real‐time sensing system for monitoring the cell states (Figure 5b). According to the sensing system, the COMA sensor can transform the deformation and temperature information of the pouch cell into one comprehensive electrical signal, enabling subsequent analysis and early failure detection.

Moreover, it is crucial to emphasize that the early‐stage signals detected by the COMA sensor play a most important role in identifying the state of the cell [40], which can remind the user to ensure safe usage. Under such conditions, it may not be essential to acquire the detailed quantitative data of battery swelling or temperature. Instead, rapid identification of the operational state of the cell is of greater significance. As a result, in Figure 5c, the output signals of the COMA sensor when the cell is under different states for the first 120 s are respectively shown. During normal charge, the resistance of the COMA sensor slightly increases and then gradually decreases to its initial state. The reason for this phenomenon points to the insertion of lithium ions into the graphite anode during the initial charging stage, which induces an increase in interlayer spacing and volume expansion, following with structure relaxation to its initial state [41, 42]. Furthermore, during a fast charge process, the higher charging voltage applied to the cell accelerates the anode reaction, leading to a more significant volume expansion, which results in a higher maximum normalized resistance of the COMA sensor. Besides the charging process, two common failure modes are further discussed. During the short circuit, the cell rapidly releases energy via Joule heat until the power of the cell is exhausted, which compromises the security. It is expected that the COMA sensor can detect the volume expansion and temperature increase simultaneously, which is vital for mitigating safety risks. To simulate thermal runaway, the pouch cell was subjected to continuous heating, mimicking exposure to external heat sources. The continuously rising resistance signal observed by the COMA sensor directly correlates with the continuous volume expansion and temperature increase, indicating the failure of the power system. These early‐stage electrical signatures enable predictive diagnostics of battery conditions, offering a promising route toward proactive safety management in wearable power systems.

To validate the feasibility of intelligent state recognition, the algorithm‐assisted analysis was employed to achieve rapid identification. The MLP algorithm was applied, and an artificial neural network (ANN) was trained to classify the four operational states based on resistance‐time sequences. To be detailed, the resistance signal was sampled at equal intervals for one second and transformed into a vector, which has 120 features correspondingly and acts as one sample of the dataset. After the data augmentation and the standardization process, the samples were partitioned into the train set (80%) and test set (20%), which contain 1600 and 400 samples, respectively. The train set was then passed into the ANN, which contains an input layer, 3 hidden layers with 128, 64, and 64 neurons, respectively, and an output layer. The ReLU function was used as the activation function of the hidden layers, and the Sigmoid function was used for the output layer. The structure diagram of the ANN above is shown in Figure 5d. To evaluate the effectiveness of the model and the training process, the cross‐entropy was used as the loss function. After 3000 epochs, the loss and the accuracy on the training set tend to be stable, as shown in Figure 5e, indicating that the training of the model is completed. To evaluate the effectiveness of the model, the accuracy of the ANN on the test set was calculated, reaching 97.0% (Figure 5f). This result demonstrates that the COMA sensor, when integrated with machine learning algorithms, serves as a reliable platform for real‐time battery state monitoring. More broadly, this synergy between material design and data‐driven analytics highlights the potential of COMA‐based systems for next‐generation intelligent and predictive sensing technologies.

## 3 Conclusion

Inspired by the bead‐chain morphology of *Nostoc*, we developed a COMA sensor fabricated through a simple unidirectionally rubbing process. The crack‐like junctions between adjacent microspheres endow the COMA sensor with high strain sensitivity, achieving a maximum gauge factor of 89. By exploiting the thermal expansion of the silicone substrate, the COMA sensor also functions as a highly responsive temperature sensor, exhibiting a maximum sensitivity of 75.2%°C^−1^ over 20°C–60°C. Furthermore, integration with the MLP algorithm enables classification of four critical battery states with an accuracy of 97.0%, highlighting its promise for intelligent and reliable battery state monitoring. In summary, the bioinspired COMA architecture offers a structurally guided route to thermomechanically stable, dual‐functional sensors. This design paradigm may inspire future intelligent soft electronics capable of adaptive, multimodal, and self‐learning perception.

## 4 Materials and Methods

### 4.1 Materials

PDMS resin (Sylgard 184) was purchased from Dow Corning Co., Ltd. Ecoflex resin (Ecoflex 00–30) was purchased from Smooth‐on Inc. PS microspheres were purchased from Suzhou Knowledge & Benefit Sphere Tech. Co., Ltd. Aniline (99.5%, after redistillation) was purchased from Sigma–Aldrich Chemical Co., Ltd. Potassium persulfate (≥99.99% metals basis) was purchased from Shanghai Aladdin Biochemical Technology Co., Ltd (China). Hydrochloric acid was obtained from Sinopharm Chemical Reagent Co., Ltd. (China).

### 4.2 Fabrication of the Groove Array Silicone Substrate

The groove array silicone substrate was prepared by the drop‐cast process with a silicon wafer customized by Suzhou YW MENS Co., Ltd. First, the PDMS base and curing agent were mixed in a mass ratio of 10:1, and the Ecoflex glue A and glue B were mixed in a mass ratio of 1:1. Then, the two uncured resins were mixed in a mass ratio of 1:1. The resin was then drop cast onto the customized silicon wafer following by a degas process. After curing in an oven at 80°C for 2 h, the groove array silicone substrate was obtained. Additionally, the silicone substrate was cut into 5 mm × 15 mm and 10 mm × 12 mm for strain sensing and temperature sensing, respectively.

### 4.3 Fabrication of the PANI@PS Microspheres

PANI@PS microspheres were synthesized through in situ polymerization. Typically, 0.2 g of PS microspheres with a diameter of 10 µm and 66.7 µL of aniline were dispersed into 2 mL of deionized water. Then 1 m hydrochloric acid was dropped into the solution until the pH was adjusted to 2 and further stirred at 300 rpm for at least 2 h. Subsequently, 0.0968 g of potassium persulfate dissolved in 10 mL of 0.05 m hydrochloric acid was added and stirred at 300 rpm in an ice‐water bath for 10 h to obtain PANI@PS microspheres. These microspheres were washed with ethanol for three times and dried in a vacuum at 40°C for 12 h.

### 4.4 Fabrication of the COMA Sensor

Two groove array silicone substrates were placed face‐to‐face along the longitudinal structure. The COMA structure was eventually obtained by unidirectional rubbing of PANI@PS microspheres along the longitudinal path. Subsequently, a PDMS encapsulation was used to protect conductive pathways, and silver ink was coated to form the electrodes.

### 4.5 Sensing Behavior Test

The resistance signal measurements of the COMA sensor were record in real‐time on a SourceMeter (2602B SYSTEM, KEITHLEY) during the loading or heating process. The voltage applied to the sample in the experiment was 2 V. The strain sensing of the COMA sensor was performed on an ElectroForce mechanical test machine (ElectroForce 3220, TA Instruments), and the test frequency of the pressure sensor cyclic measurement was 1 Hz. The temperature sensing of the COMA sensor was performed on a TEC temperature controller (TCM‐X107, Chengdu Yexian Tech. Co., Ltd.). The battery state monitoring was performed on lithium‐ion polymer batteries (084546, Guangdong Yaner Industrial Co., Ltd.), and the COMA sensor was attached to the surface of the pouch cell by using commercial thin double‐faced adhesive tape. During the normal charge and fast charge processes, the cell was charged with a voltage of 4 and 4.5 V, respectively. During the thermal runaway simulation, the cell was continuously heated at 60°C with a heating platform.

### 4.6 Characterization and Measurements

The morphologies of the silicone substrate, the PS microspheres, and the PANI@PS microspheres were characterized by SEM (Ultra 55, Zeiss). The three‐dimensional graph of the silicone substrate was characterized by LSCM (LSM 900, Zeiss). Mechanical properties of PDMS, Ecoflex, silicone substrate, and commercial PET tape were tested on a material mechanics test instrument (Instron 5966). The CTE of PDMS, Ecoflex, and the silicone substrate were tested on a thermal mechanical analyzer (SDTA2+, Mettler Toledo) with a heating rate of 3°C/min and a preload of 0.02 N.

### 4.7 Data Collection and Analysis for Machine Learning

During data preprocessing, the signal waveform was first converted into a vector based on a fixed time interval, forming the raw data set. To address the non‐reproducibility of abnormal states, data augmentation was performed by adding Gaussian‐distributed errors to each vector, with a mean of 0.3 times the standard deviation of the original data. All the data were integrated into one data set and then standardized by normalizing each vector to have a mean of 0 and a standard deviation of 1. Finally, after partition, the train set and the test set contain 80% and 20% samples, respectively. The MLP algorithm was chosen to classify four different cell states. To improve the stability and robustness of the model, the cross‐entropy is used as the loss function to evaluate the effectiveness of the model, and the Stochastic Gradient Descent (SGD) was chosen as the optimizer.

## Author Contributions

W.X. equally contributed to the conceptualization, data curation, formal analysis, investigation, methodology, software, validation, visualization, writing – original draft, and writing – review and editing. H.T. worked on investigation, methodology, supervision, and validation. Z.W. contributed to investigation, validation, and visualization. T.Z. worked on methodology, validation, and visualization. C.W. contributed to methodology, and validation. M.C. was responsible for conceptualization, funding acquisition, project administration, and writing – review and editing. L.S. contributed to conceptualization, data curation, formal analysis, funding acquisition, investigation, project administration, resources, supervision, and writing – review and editing. L.W. worked on conceptualization, funding acquisition, project administration, resources, supervision, and writing – review and editing.

## Funding

Financial support for this research from the National Key Research and Development Program of China (2022YFA1205200) and the National Natural Science Foundation of China (52403086, 51721002, and 52033003) is acknowledged.

## Conflicts of Interest

The authors declare no conflicts of interest.

## Supporting information




**Supporting File**: advs75452‐sup‐0001‐SuppMat.docx.

## Data Availability

The datasets generated during and/or analyzed during the current study are available from the corresponding author.
