# Supplementary material for: A Crack‐Based One‐Dimensional Microspheres Array Enables Thermal–Mechanical Decoupled Dual‐Functional Sensing
Source: Adv Sci (Weinh). 2026 Apr 27;13(41):e75452. doi: 10.1002/advs.75452 (PMC13335698; doi:10.1002/advs.75452)
Supplement: Supplementary file 1 — Supporting File: advs75452‐sup‐0001‐SuppMat.docx. [file ADVS-13-e75452-s001.docx]

Supporting information

**A Crack-Based One-Dimensional Microspheres Array Enables Thermal–Mechanical Decoupled Dual-Functional Sensing**

*Wanqing Xu^a^, Hongyi Tu^a^, Zehao Wang^a^, Tianyu Zhu^a^, Chao Wang^a^, Min Chen^a^, Lan Shi^a^ *, Limin Wu^a^*

a. College of Smart Materials and Future Energy, State Key Laboratory of Coatings for Advanced Equipment, Advanced Coatings Research Center of Ministry of Education of China, Fudan University, Shanghai 200433, China

*Corresponding author. E-mail: sl@fudan.edu.cn (L. S.)

**Supplementary Note S1.** The theoretical model of the COMA sensor.

As shown in Figure S9a, the sensor is composed of *k* rows of PANI@PS microspheres, and the number of PANI@PS microspheres in each row is $n_{i}$.

For simplicity, it is approximately assumed that the number of PANI@PS microspheres on each row is equal, which means:

$$\begin{aligned} n_{1}=n_{2}=\cdots=n_{i}=n Eq.1 \end{aligned}$$

On this basis, the sensor model can be abstracted as shown in Figure S9b, indicating that the sensor is composed of *k* resistors connected in parallel. Each resistor corresponds to a row of microspheres. Among them, $R_{s}$ represents the resistance value of each microsphere and $R_{c}$ represents the contact resistance between two microspheres.

As a result, the resistance value $R_{i}$ of each row of resistors can be expressed as:

$$\begin{aligned} R_{i}=n\cdot R_{s}+\left( n-1 \right)\cdot R_{c} Eq.2 \end{aligned}$$

For the entire sensor, in the initial state, $R_{0}$ is a parallel connection of *k* resistors, that is

$$\begin{aligned} R_{0}=R_{1}\parallel R_{2}\parallel\cdots\parallel R_{k}=\frac{R_{i}}{k}=\frac{n\cdot R_{s}+\left( n-1 \right)\cdot R_{c}}{k} Eq.3 \end{aligned}$$

For $R_{s}$, it should be related to the ambient temperature, which can be expressed by the following formula:

$$\begin{aligned} R_{s}\left( T \right)=R_{s}\cdot\left( 1+TCR\cdot\left( T-T_{0} \right) \right) Eq.4 \end{aligned}$$

where TCR is the temperature coefficient of resistance of the PANI.

For $R_{c}$, according to the previous reference,^[1]^ it should be related to the strain ($\varepsilon$) of the structure, which can be expressed by the following formula:

$$\begin{aligned} R_{c}\left( \varepsilon\right)=R_{c}\cdot e^{A\varepsilon} Eq.5 \end{aligned}$$

where *A* is an exponential factor related to property of the sensing system.

Thus, combining the effects of temperature and strain, the resistance of the sensing system should be expressed as follow:

$$\begin{aligned} R\left( T, \varepsilon\right)=\frac{n\cdot R_{s}\left( T \right)+\left( n-1 \right)\cdot R_{c}\left( \varepsilon\right)}{k} \\ =\frac{n\cdot R_{s}\cdot\left( 1+TCR\cdot\left( T-T_{0} \right) \right)+\left( n-1 \right)\cdot R_{c}\cdot e^{A\varepsilon}}{k} Eq.6 \end{aligned}$$

Therefore, the output signal is as follow:

$$\frac{\Delta R}{R_{0}}=\frac{R\left( T,\varepsilon\right)-R_{0}}{R_{0}}=\frac{\frac{n\cdot R_{s}\left( T \right)+\left( n-1 \right)\cdot R_{c}\left( \varepsilon\right)}{k}-\frac{n\cdot R_{s}+\left( n-1 \right)\cdot R_{c}}{k}}{\frac{n\cdot R_{s}+\left( n-1 \right)\cdot R_{c}}{k}}$$

$$\begin{aligned} =\frac{n\cdot\left( R_{s}\left( T \right)-R_{s} \right)+\left( n-1 \right)\cdot\left( R_{c}\left( \varepsilon\right)-R_{c} \right)}{n\cdot R_{s}+\left( n-1 \right)\cdot R_{c}} Eq.7 \end{aligned}$$

It should be noticed that the PANI is a conductor with a relatively low resistivity. Moreover, the TCR of the PANI should be less than 0.02 °C^-1^ according to the previous reference.^[2]^ According to the properties of functions, it is rational to assume that

$$\begin{aligned} R_{s}\cdot\left( 1+TCR\cdot\left( T-T_{0} \right) \right)\ll R_{c}\cdot e^{A\varepsilon} Eq.8 \end{aligned}$$

The result mentioned above indicates that the resistance of the sensor system is dominated by the contact resistance between two microspheres instead of the resistance of the PANI@PS microspheres.

Moreover, since *n* is a large value (approximately 500 in one array), *n* is approximately equal to *n*-1. Thus, for the strain sensing, the output signal is as follow:

$$\begin{aligned} \frac{\Delta R}{R_{0}}=\frac{n\cdot\left( R_{s}\left( T \right)-R_{s} \right)+\left( n-1 \right)\cdot\left( R_{c}\left( \varepsilon\right)-R_{c} \right)}{n\cdot R_{s}+\left( n-1 \right)\cdot R_{c}}\approx\frac{R_{c}\left( \varepsilon\right)-R_{c}}{R_{c}}=e^{A\varepsilon}-1 Eq.9 \end{aligned}$$

Moreover, for the temperature sensing, the strain is only provided by the thermal expansion of the substrate. The output signal is as follow:

$$\begin{aligned} \frac{\Delta R}{R_{0}}=\frac{R\left( \varepsilon\right)-R_{0}}{R_{0}}=e^{A\varepsilon}-1=e^{A\cdot CTE\cdot\left( T-T_{0} \right)}-1 Eq.10 \end{aligned}$$

where CTE is the coefficient of thermal expansion of the silicone substrate.


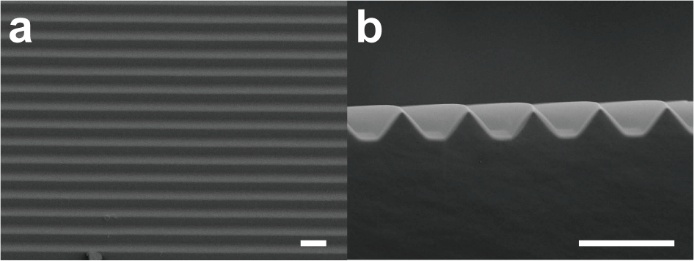


**Figure S1.** SEM images of the groove array silicone substrate (a) Top view. (b) Side view. Scale bar, 20 μm.


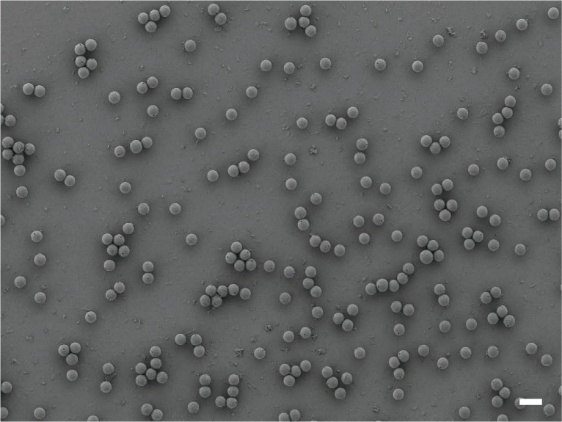


**Figure S2.** SEM images of the monodispersed PS microspheres. Scale bar, 20 μm.


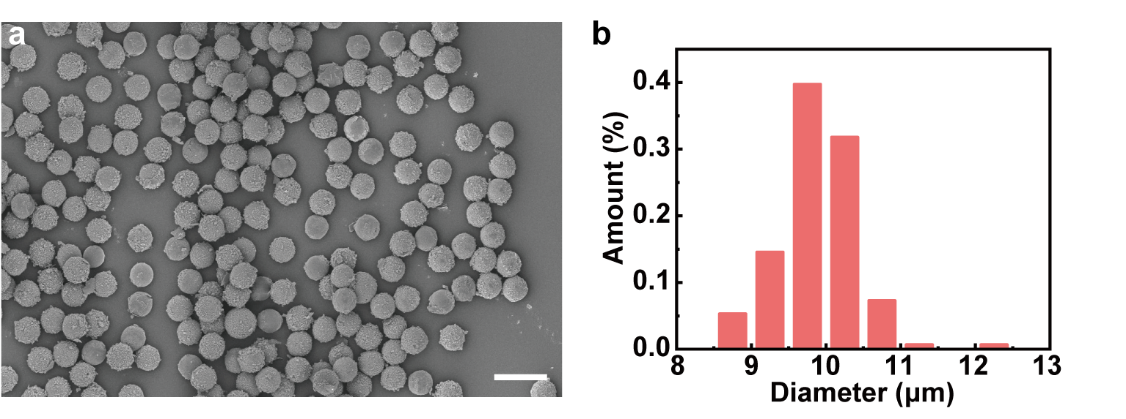


**Figure S3.** (a) SEM images of the PANI@PS microspheres. Scale bar, 20 μm. (b) Size distribution of the microspheres.


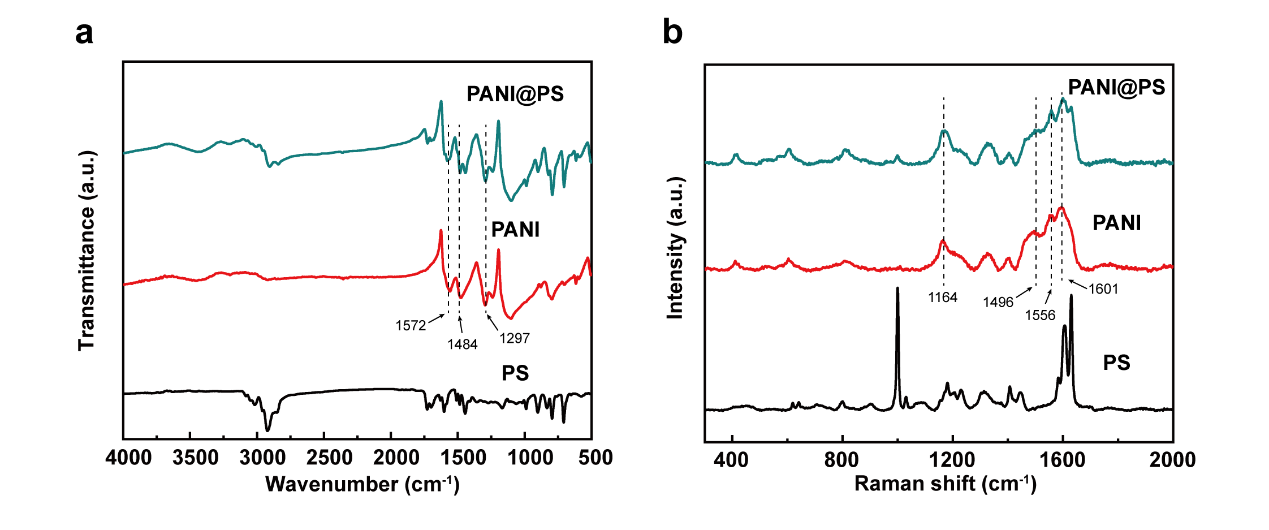


**Figure S4.** Chemical structure characterization of PANI, PS and PANI@PS. (a) FT-IR (b) Raman spectrum.


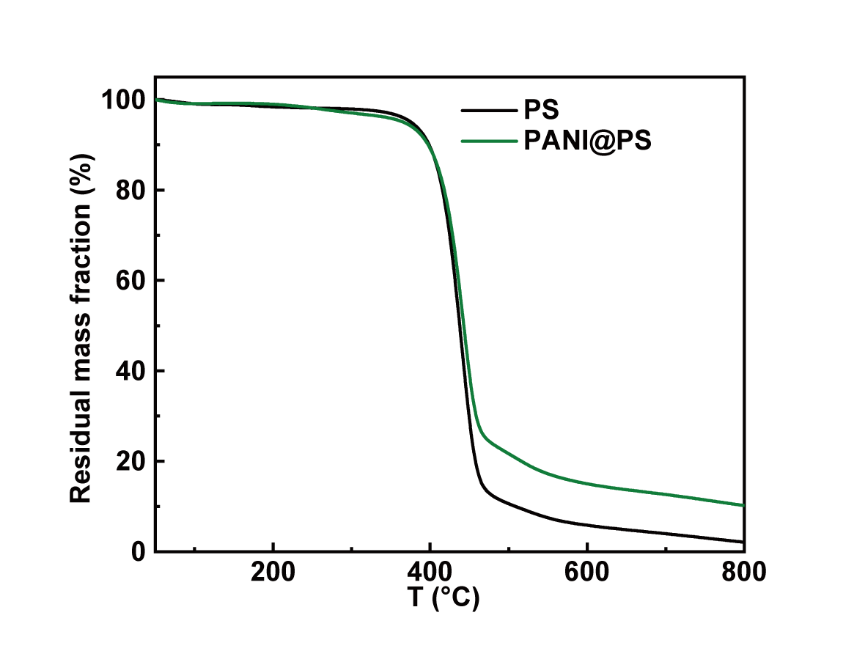


**Figure S5.** TGA of PS and PANI@PS.


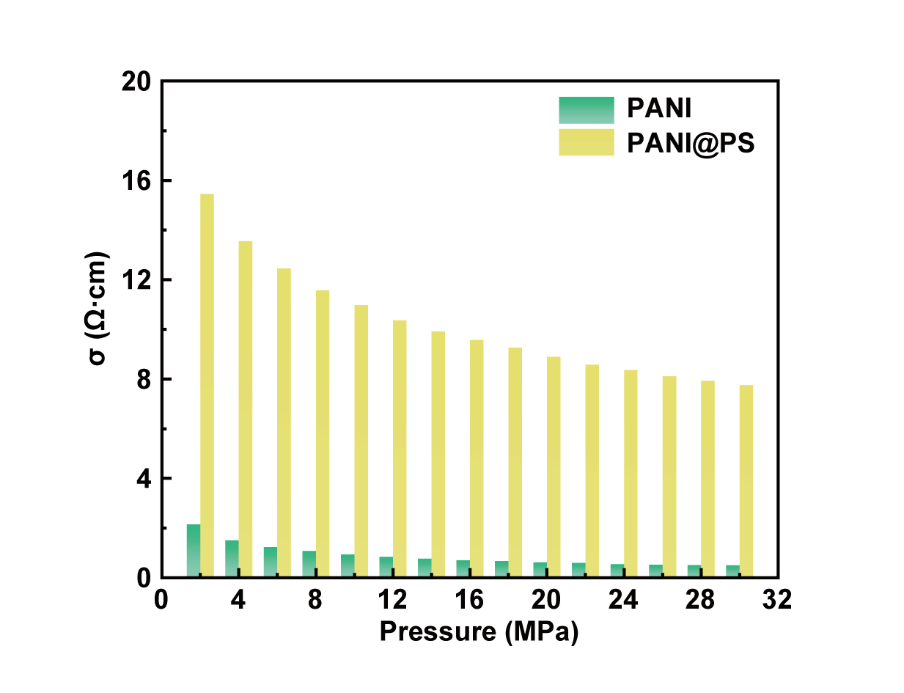


**Figure S6.** The electrical resistivity of PANI and PANI@PS under different pressure.


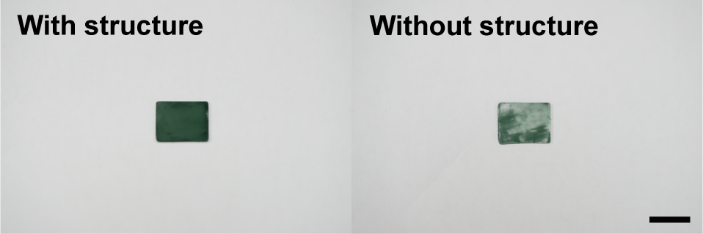


**Figure S7.** The optical graphs of the adhesion condition of PANI@PS microspheres on the silicone substrates with and without the microstructure of grooves. Scale bar, 1cm.


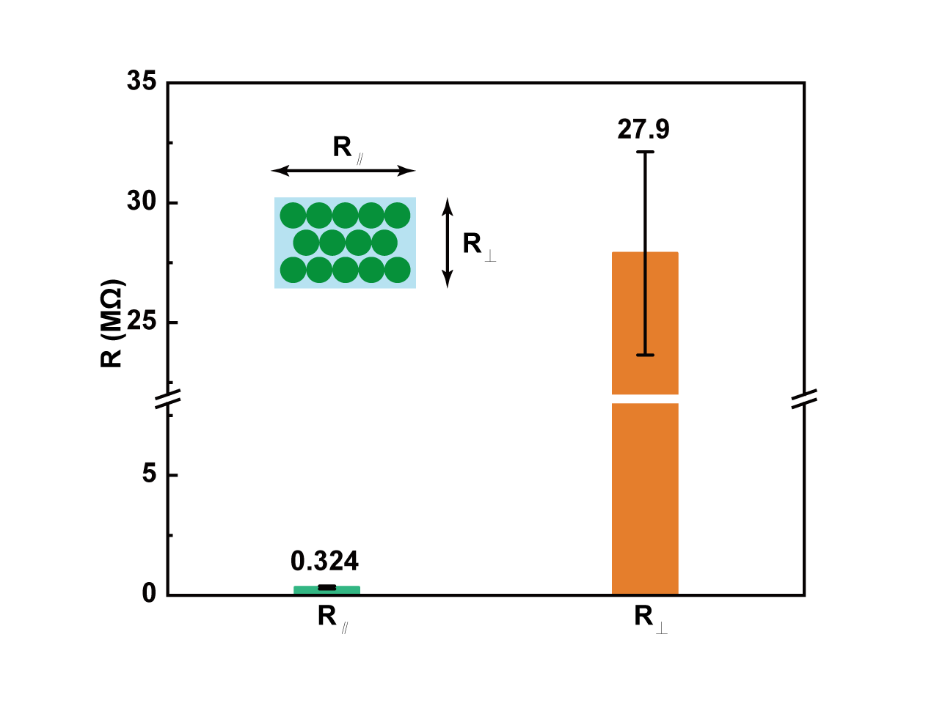


**Figure S8.** Comparison of the resistance in parallel and perpendicular directions to the grooves.


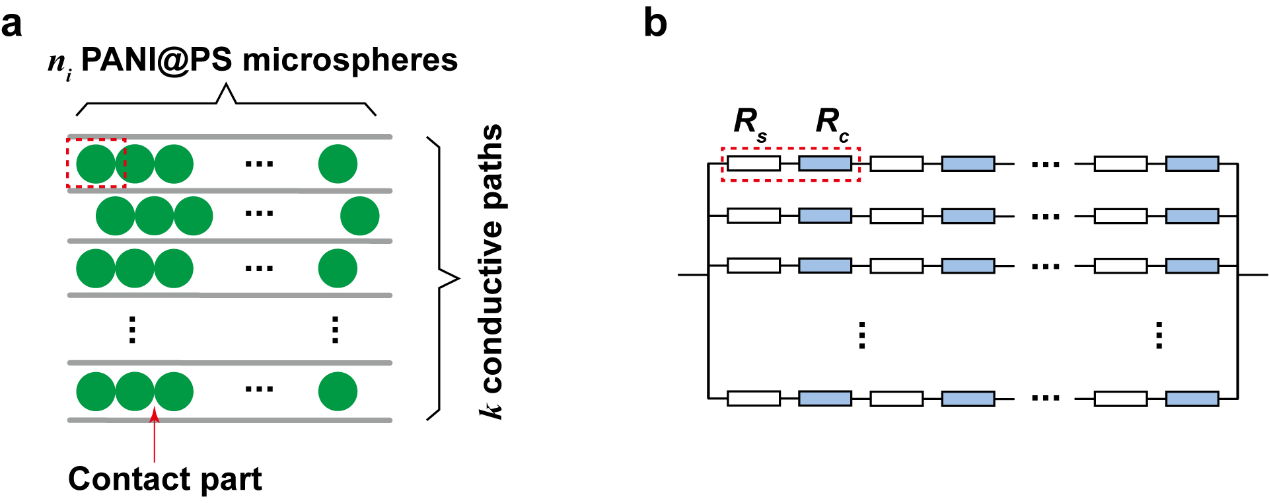


**Figure S9.** (a) Schematic diagram of the COMA sensor. (b) The ideal connection state of the resistors of the COMA sensor.


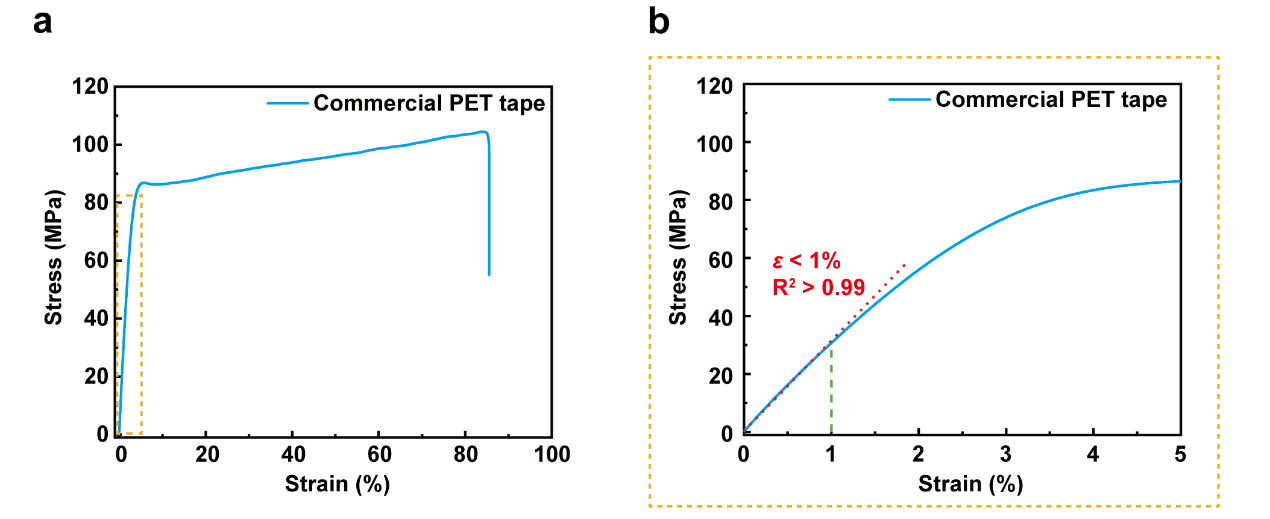


**Figure S10.** (a) The stress-strain curve of the commercial PET tape. (b) The detail of the curve when the strain is less than 5%.


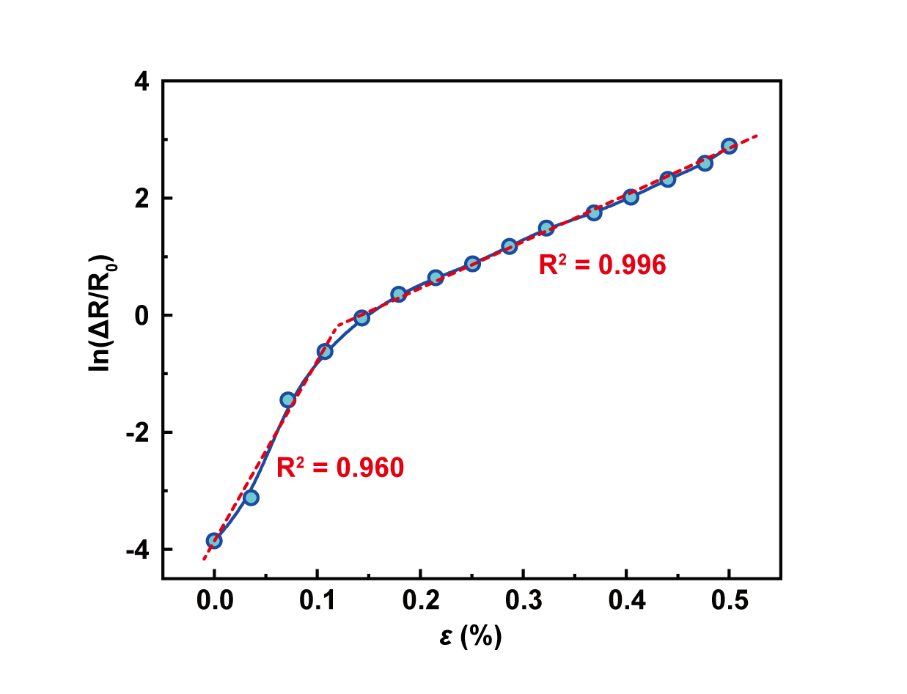


**Figure S11.** Fitting lines for logarithmic normalized resistance according to strain.


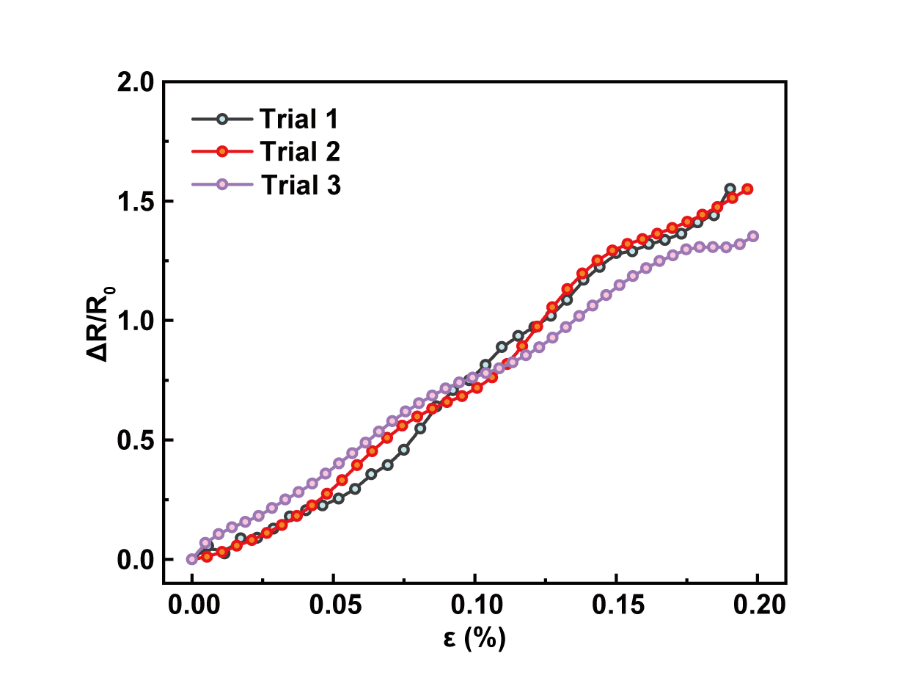


**Figure S12.** Consistency of the COMA sensor during different stretching processes with a maximum strain of 0.2%.


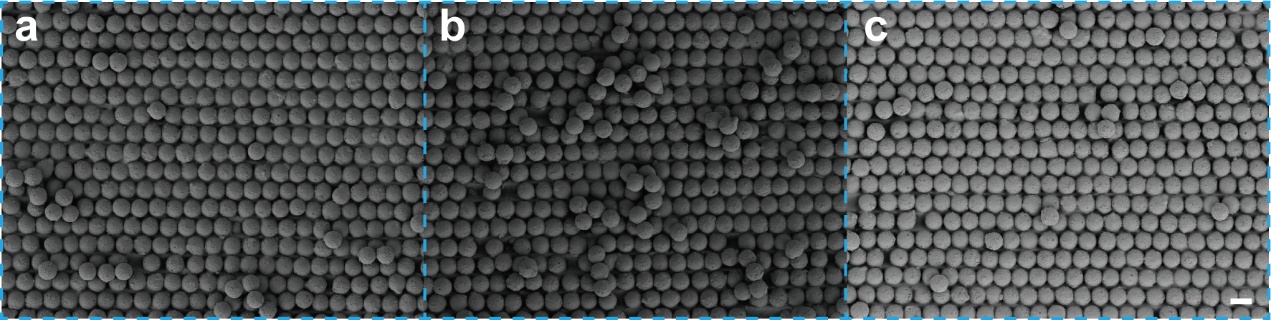


**Figure S13.** The SEM images of the COMA sensor (a) in the initial state (b) after cyclic loading-unloading for 1,000 cycles with a maximum strain of 0.3% (c) after heating at 60 °C for 1 hour. Scale bar, 10 μm.


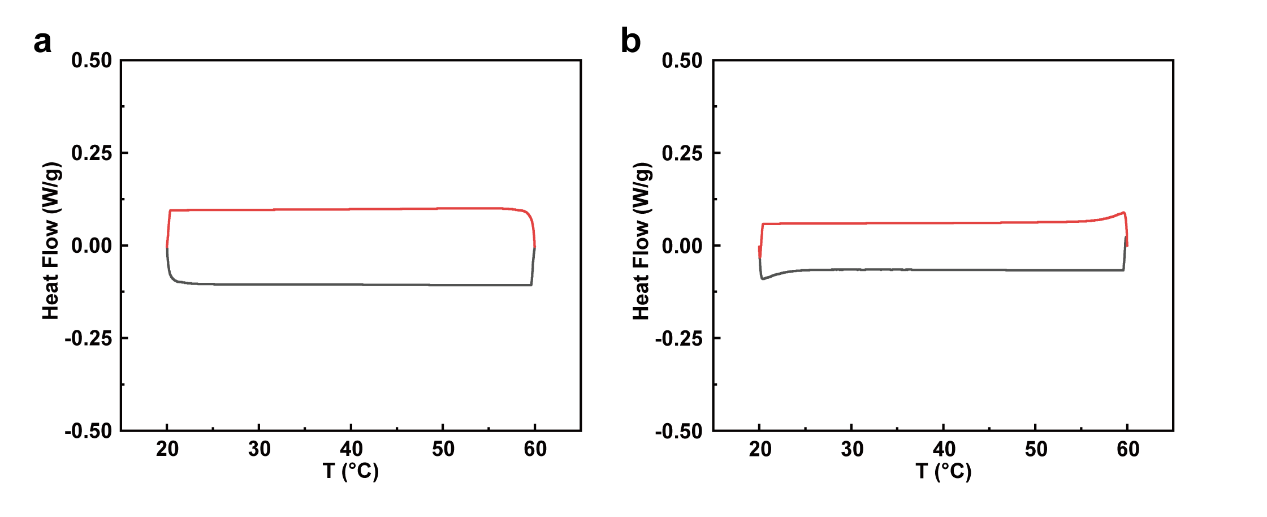


**Figure S14.** DSC thermograms of (a) Silicone substrate and (b) PANI@PS microspheres in the temperature range of 20-60 °C


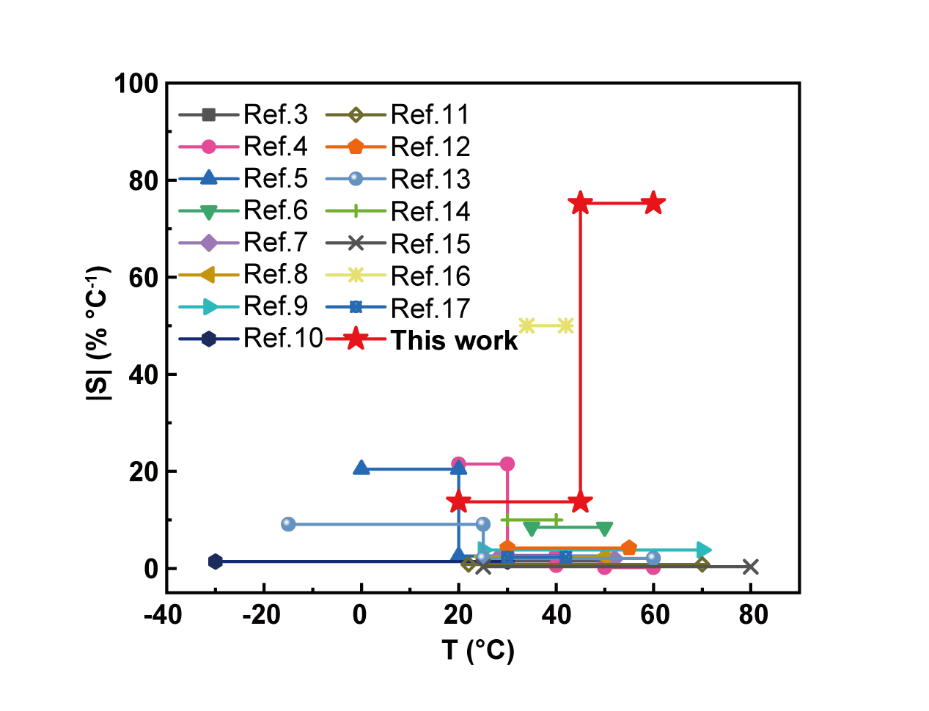


**Figure S15.** Comparison of the COMA sensor performance on temperature sensing with previous literature work.


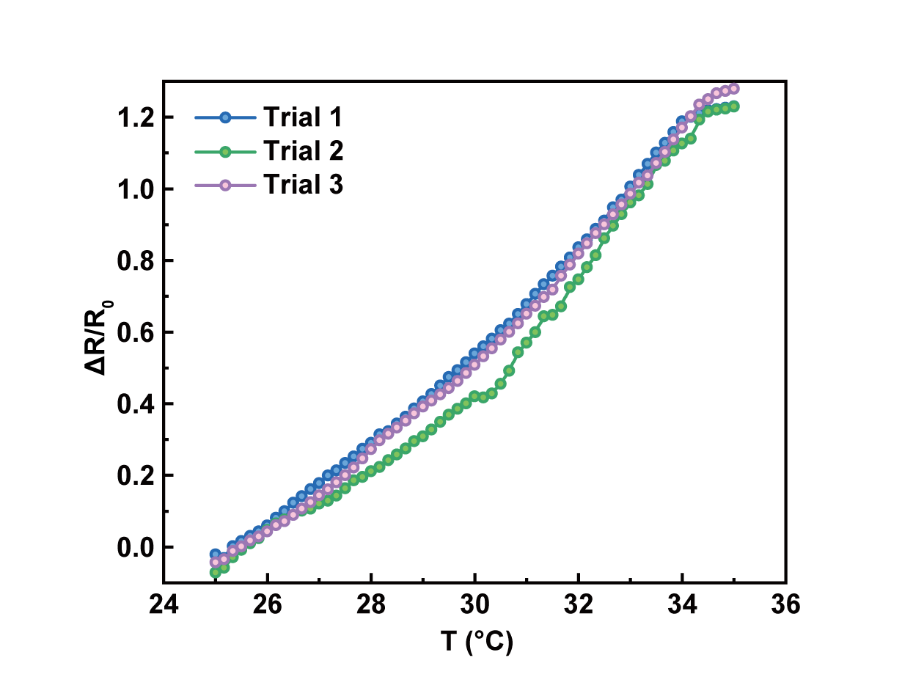


**Figure S16.** Consistency of the COMA sensor during different heating processes within the range of 25-35 °C.


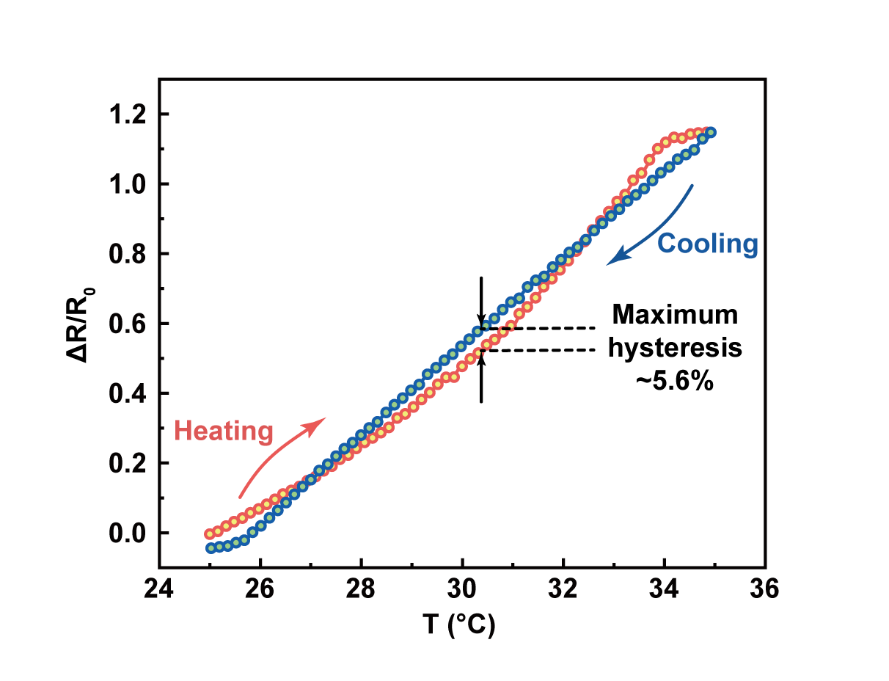


**Figure S17.** The hysteretic curve of the COMA sensor within the range of 25-35 °C.


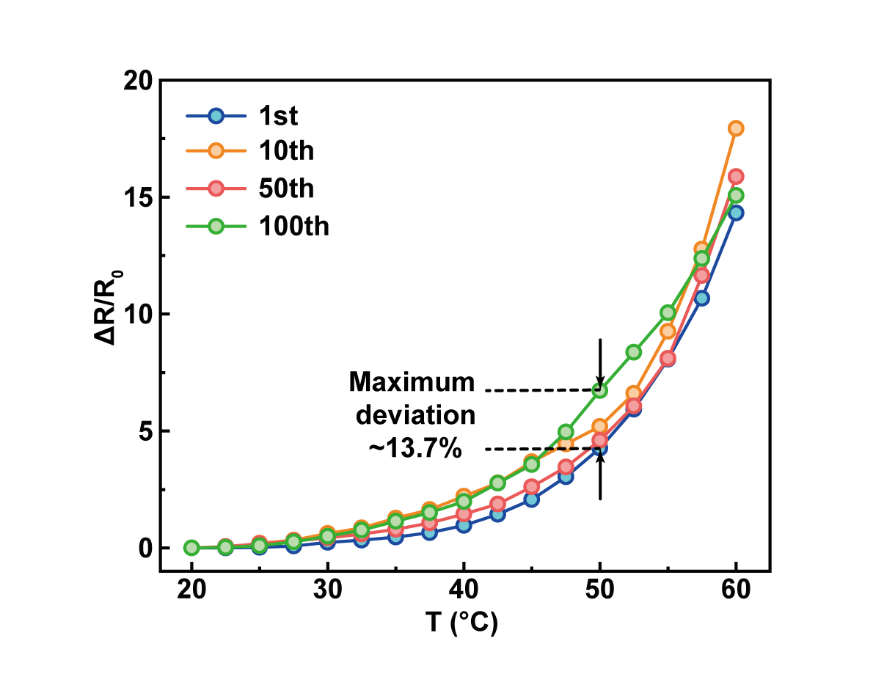


**Figure S18.** The repeatability of the COMA sensor after different numbers of heating cycles from 20 to 60 °C.


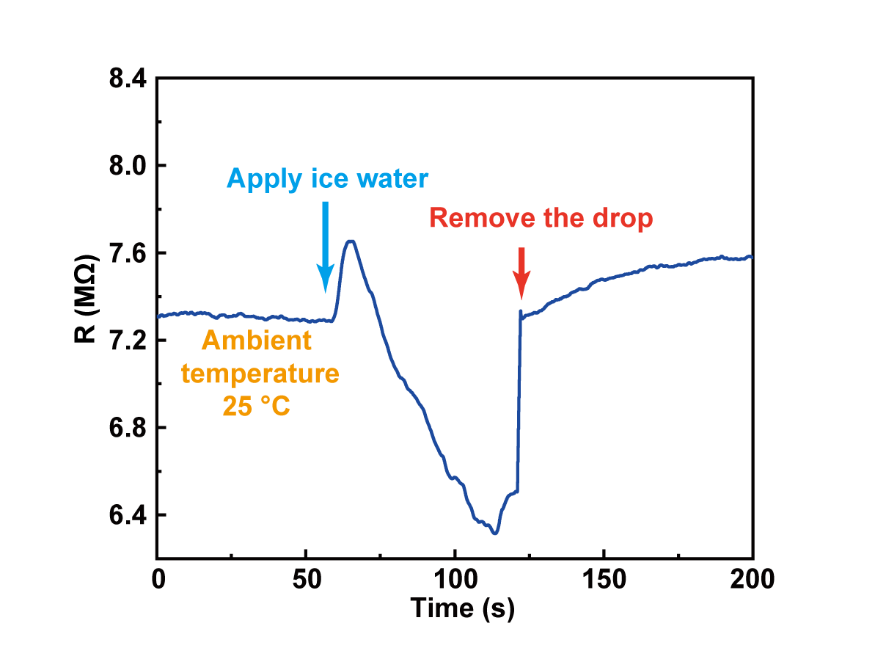


**Figure S19.** The performance of the COMA sensor applied with ice water.


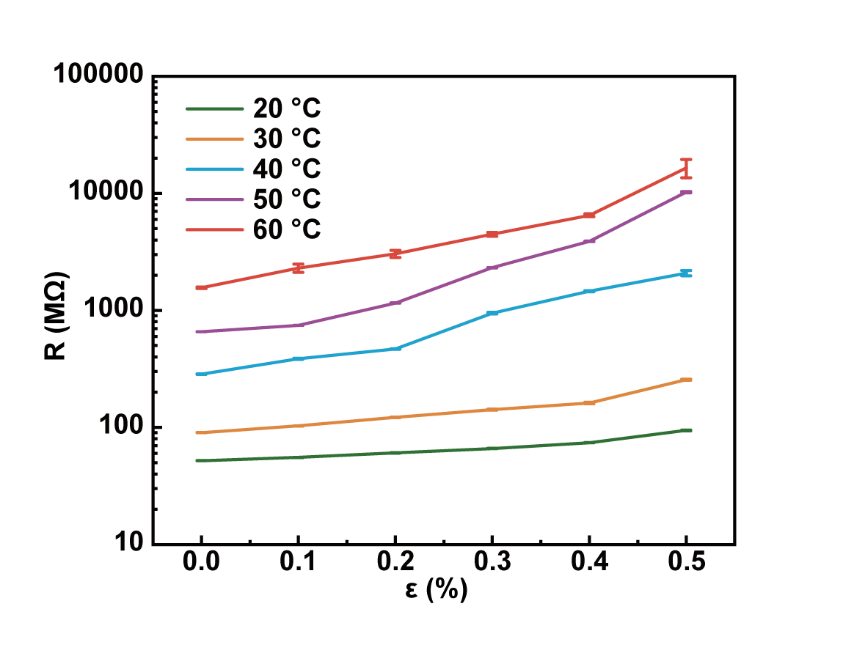


**Figure S20.** The resistance changes of the COMA sensor during the stretching process at different adjacent temperatures.


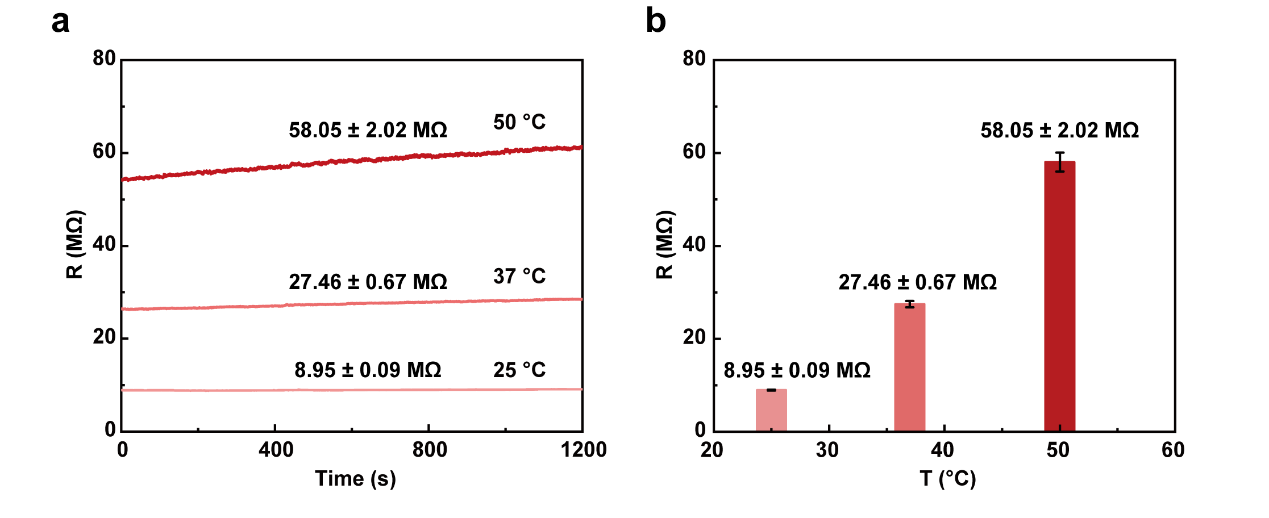


**Figure S21.** (a) The fluctuation of resistance of the COMA sensor during long heating for 1200 s. (b) The statistical results of resistance under different temperatures.**Table S1.** The comparison of the temperature sensing performance of the COMA sensor and previous literature work.

| **Material** | **Sensitivity (% °C^-1^)** | **Range (°C)** | **Reference** |
| --- | --- | --- | --- |
| PU/CNT/PEDOT:PSS fiber | -1.53 | 30-50 | [3] |
| rGO/PEO/PVDF | 21.5  2.67  0.606  0.192 | 20-30  30-40  40-50  50-60 | [4] |
| Fe_3_O_4_@C/gelatin hydrogel | -20.44  -2.54 | 0-20  20-50 | [5] |
| Phase-transformable ionic conductors | -8.50 | 35-50 | [6] |
| PAM/CAR/Gly hydrogel | -2 | 28-52 | [7] |
| CNF Film | -2.44 | 25-50 | [8] |
| Na^+^-SA xerogel | -3.77 | 25-70 | [9] |
| PEDOT:PSS/CNT/Ecoflex | 1.41 | -30-30 | [10] |
| CNT/PDMS | 0.83 | 22-70 | [11] |
| PEDOT:PSS/PDMS | 4.2 | 30-55 | [12] |
| NiO/CNT fiber | -9.1  -2.1 | -15-25  25-60 | [13] |
| MXene/PEO aerogel | -10 | 30-40 | [14] |
| Au/PDMS | 0.32 | 25-80 | [15] |
| PEDOT:PSS/MXene/PVA organic hydrogel | 50 | 34-42 | [16] |
| Mxene/PEDOT:PSS/CMC | -2.3275 | 30-42 | [17] |
| **PANI@PS/PDMS-Ecoflex** | **13.7**  **75.2** | **20-45**  **45-60** | **This work** |

**Reference**

[1] D. Kang, P. V. Pikhitsa, Y. W. Choi, C. Lee, S. S. Shin, L. Piao, B. Park, K.-Y. Suh, T.-i. Kim, M. Choi, *Nature* **2014**, *516* (7530), 222.

[2] H. Liu, K. Sun, X.-L. Guo, Z.-L. Liu, Y.-H. Wang, Y. Yang, D. Yu, Y.-T. Li, T.-L. Ren, *ACS Nano* **2022**, *16* (12), 21527.

[3] Y. Yin, C. Guo, Q. Mu, W. Li, H. Yang, Y. He, *Chem. Eng. J.* **2024**, *500*, 157115.

[4] Y. Lee, J. Park, A. Choe, Y.-E. Shin, J. Kim, J. Myoung, S. Lee, Y. Lee, Y.-K. Kim, S. W. Yi, J. Nam, J. Seo, H. Ko, *ACS Nano* **2022**, *16* (1), 1208.

[5] H. Zhang, H. M. Chen, J. H. Lee, E. Kim, K. Y. Chan, H. Venkatesan, M. H. Adegun, O. G. Agbabiaka, X. Shen, Q. B. Zheng, J. L. Yang, J. K. Kim, *Adv. Funct. Mater.* **2022**, *32* (47), 2208362.

[6] Y. Zhou, C. Yu, X. Zhang, Y. Zheng, B. Wang, Y. Bao, G. Shan, H. Wang, P. Pan, *Adv. Mater.* **2024**, *36* (15), 2309568.

[7] L. Luo, Z. Wu, Q. Ding, H. Wang, Y. Luo, J. Yu, H. Guo, K. Tao, S. Zhang, F. Huo, J. Wu, *ACS Nano* **2024**, *18* (24), 15754.

[8] J. H. Lee, E. Kim, H. Zhang, H. M. Chen, H. Venkatesan, K. Y. Chan, J. Yang, X. Shen, J. L. Yang, S. Jeon, J. K. Kim, *Adv. Funct. Mater.* **2022**, *32* (10), 2107570.

[9] G. Kumar, S. Panda, *Carbohydr. Polym.* **2024**, *340*, 122258.

[10] B. H. Liang, B. F. Huang, J. K. He, R. L. Yang, C. C. Zhao, B. R. Yang, A. Y. Cao, Z. K. Tang, X. C. Gui, *Nano Res.* **2022**, *15* (4), 3614.

[11] G. Y. Bae, J. T. Han, G. Lee, S. Lee, S. W. Kim, S. Park, J. Kwon, S. Jung, K. Cho, *Adv. Mater.* **2018**, *30* (43), 1803388.

[12] Y. Yu, S. Peng, P. Blanloeuil, S. Wu, C. H. Wang, *ACS Appl. Mater. Inter.* **2020**, *12* (32), 36578.

[13] Y. Lu, H. Zhang, Y. Zhao, H. Liu, Z. Nie, F. Xu, J. Zhu, W. Huang, *Adv. Mater.* **2024**, *36* (18), 2310613.

[14] J. Wu, X. Fan, X. Liu, X. Ji, X. Shi, W. Wu, Z. Yue, J. Liang, *Nano Lett.* **2022**, *22* (11), 4459.

[15] S. Shi, P. Zhao, P. Yang, L. Zhao, J. Yi, Z. Wang, S. Yu, *Nanomaterials* **2025**, *15* (13), 1001.

[16] P. Wang, G. Wang, G. Sun, C. Bao, Y. Li, C. Meng, Z. Yao, *Nano-Micro Lett.* **2025**, *17* (1), 156.

[17] X. Zhu, Y. Zhao, Z. Zhou, A. Zhang, W. Ma, H. Liu, Z. Lin, S. Yang, Y. Fang, X. Kong, Y. Zhongling, W. Hong, Q. Hong, X. Guo, *J. Colloid Interf. Sci.* **2025**, *699*, 138291.
